# The CAIRO4 study: the role of surgery of the primary tumour with few or absent symptoms in patients with synchronous unresectable metastases of colorectal cancer – a randomized phase III study of the Dutch Colorectal Cancer Group (DCCG)

**DOI:** 10.1186/1471-2407-14-741

**Published:** 2014-10-02

**Authors:** Jorine ’t Lam - Boer, Linda Mol, Cornelis Verhoef, Anton F J de Haan, Mette Yilmaz, Cornelis J A Punt, Johannes H W de Wilt, Miriam Koopman

**Affiliations:** Department of Surgery, Radboud university medical center, PO Box 9101, 6500 HB Nijmegen, The Netherlands; Clinical Trial Centre, comprehensive cancer centre The Netherlands, Nijmegen, The Netherlands; Division of Surgical Oncology, Erasmus MC Cancer Institute, Rotterdam, The Netherlands; Department of Epidemiology, Radboud university medical center, Nijmegen, The Netherlands; Department of Oncology, Aalborg University Hospital, Aalborg, Denmark; Department of Medical Oncology, Academic Medical Center, Amsterdam, The Netherlands; Department of Medical Oncology, University Medical Center Utrecht, Utrecht, The Netherlands

**Keywords:** Stage IV colorectal cancer, Unresectable metastases, Synchronous metastases, Palliative chemotherapy, Bevacizumab, Primary tumour, Surgical resection

## Abstract

**Background:**

There is no consensus regarding resection of the primary tumour with few or absent symptoms in patients with synchronous unresectable metastatic colorectal cancer (CRC). A potential benefit of resection of the primary tumour is to prevent complications of the primary tumour in later stages of the disease. We here propose a randomized trial in order to demonstrate that resection of the primary tumour improves overall survival.

**Methods/design:**

The CAIRO4 study is a multicentre, randomized, phase III study of the Dutch Colorectal Cancer Group (DCCG). Patients with synchronous unresectable metastases of CRC and few or absent symptoms of the primary tumour are randomized 1:1 between systemic therapy only, and resection of the primary tumour followed by systemic therapy. Systemic therapy will consist of fluoropyrimidine-based chemotherapy in combination with bevacizumab. The primary objective of this study is to determine the clinical benefit in terms of overall survival of initial resection of the primary tumour. Secondary endpoints include progression free survival, surgical morbidity, quality of life and the number of patients requiring resection of the primary tumour in the control arm.

**Discussion:**

The CAIRO4 study is a multicentre, randomized, phase III study that will assess the benefit of resection of the primary tumour in patients with synchronous metastatic CRC.

**Trial registration:**

The CAIRO4 study is registered at clinicaltrials.gov (NCT01606098)

## Background

Colorectal cancer (CRC) is the third most common type of cancer in the Netherlands, with an incidence of more than 13,000 new cases in 2011 [1]. Due to the improvement in living standard and the aging of the population, the incidence of CRC is increasing. Approximately 20% of patients with CRC present with synchronous metastases (stage IV disease, according to TNM-classification) [2]. Although curative surgery, with resection of both the primary tumour and all metastases, is an option in some patients, the majority of patients with stage IV disease end up in a palliative setting.

Median survival in patients with advanced CRC without any form of treatment is estimated at 8 months [3]. Palliative systemic treatment, consisting of cytotoxic chemotherapy and targeted therapy, can lead to a significant benefit in overall survival [4]. Standard of care in the Netherlands in first-line treatment consists of fluoropyrimidine-based chemotherapy in combination with bevacizumab [5].

To prevent complications from the primary tumour, such as obstruction or bleeding, or to reduce symptoms, resection of the primary tumour is often considered in this patient group. Retrospective data show that approximately 50% of all patients with stage IV disease undergo resection of the primary tumour [2, 6]. The 30-day mortality rates after surgery of the primary tumour for patients with stage IV disease range between 1.3-11.7%, which is higher than reported for elective surgery in stage I-III patients [7]. However, these rates usually reflect both symptomatic and asymptomatic patients with a variety in age and comorbidity. Limited postoperative survival is furthermore associated with an extensive hepatic tumour load, pT4 tumours, lymphatic spread and R1-2 resection [8].

The indication of palliative resection prior to initiation of systemic treatment in patients with a symptomatic primary tumour is obvious. However, in patients with few or absent symptoms the indication for prophylactic resection is under debate, and its effect on survival and quality of life is still uncertain [9]. Currently, there are no data from prospective randomized trials to assess the value of resection of the primary tumour in stage IV patients with mild or absent symptoms of their primary tumour. Retrospective analysis does not provide definitive answers, since usually no reliable information is provided on the presence of symptoms at diagnosis or the indication to perform or to refrain from resection of the primary tumour. Most randomized studies in metastatic CRC do not even report whether or not a resection of the primary tumour has been performed [10].

Treatment in patients with unresectable metastatic CRC should be based on two objectives: first, to improve or maintain the quality of life, and, secondly, to prolong the survival. In patients with few or absent symptoms of the primary tumour, arguments both in favour and against initial resection have risen.

The main argument against resection of the tumour is that survival benefit of initial resection of the primary tumour has not yet been investigated in a prospective randomized trial. Therefore, surgery-related morbidity and mortality should be avoided [11–13]. Furthermore, there is some evidence from preclinical and clinical data showing that resection of the primary tumour may have a stimulating effect on the growth of distant metastases [14]. However, these data are mainly derived from in vitro and animal models and it remains unknown if and how they influence overall survival and quality of life. Also, it is argued that systemic treatment can safely be administered without prior resection of the primary tumour [15]. Thus, life-prolonging systemic treatment would only be postponed when it is decided to perform a surgical intervention first [15–17]. Additionally, Poultsides *et al.* demonstrate that most patients with synchronous stage IV CRC who receive upfront systemic therapy never require palliative surgery [15]. However, the median overall survival time in this study was only 13 months, whereas median overall survival times of 20–24 months have consistently been reported in the general population of metastatic CRC patients. Lastly, in 70% of patients who received systemic treatment prior to resection of the primary tumour major histological tumour regression was observed, suggesting that initial chemotherapy can control the primary tumour in the majority of patients [18].

The main argument in favour of resection is that it will prevent possible complications of the primary tumour, such as bleeding, obstruction or perforation [19–21]. Patients who receive initial systemic therapy without prior resection of the primary tumour are more likely to develop complications of the primary tumour [22]. Furthermore, surgery can lead to more accurate staging of disease, as extrahepatic metastases, particularly in the peritoneal cavity, may be better identified by visual exploration.

Retrospective analysis of two large randomized trials in patients with advanced CRC demonstrated that survival of patients with synchronous advanced CRC was significantly higher in patients who underwent resection of the primary tumour prior to study treatment, compared to patients with the primary tumour in situ (20.7 vs. 13.4 months, respectively) [23]. Symptoms which might be related to the primary tumour, such as nausea, vomiting and ileus did occur more often in the non-resection group. However, selection bias cannot be excluded, as the decision whether or not a patient would undergo resection was made prior to study entry. Therefore no data are available on patient characteristics that might have influenced this decision, such as resectability and symptomatology of the primary tumour and condition of the patient.

In summary, the available literature does not provide an outright support for either of the two strategies, although most support seems to exist for surgery of the primary first (Table 1). We therefore propose a prospective trial that will help provide an answer to the question which strategy is to be preferred.

**Table 1:** **Data on resection versus non-resection of the primary tumour in metastatic CRC patients**

Study	Years of study	Number of patients		Median survival time (in months)		p-value
		**Resection**	**Non-resection**	**Resection**	**Non-resection**	
**Liu** [20]	1986-1991	57	5	11	2	n.a.
**Ruo** [24]	1996-1999	127	103	16	9	<0.001
**Kaufman** [25]	1998-2003	115	69	22	3	<0.0001
**Scoggins** [13]	1985-1997	66	23	14.5	16.6	0.59
**Tebbutt** [26]	1990-1999	280	82	14	8.2	0.08
**Michel** [27]	1996-1999	31	23	21	14	0.718
**Benoist** [16]	1997-2002	32	27	23	22	n.a.
**Evans** [28]	1999-2006	45	57	11	2	< 0.0001
**Poultsides** [15]	2000-2006	-	178	-	13	
**Venderbosch** [23]	2003-2004	258	141	16.7	11.4	< 0.0001
	2005-2006	289	159	20.7	13.4	<0.0001

n.a. = not applicable.

## Methods/design

### Hypothesis

Although literature does not provide an outright support for either of the two strategies, most retrospective data seem to favour surgery of the primary tumour followed by systemic therapy over systemic therapy alone. Obviously, in patients with few or absent symptoms of their primary tumour, surgery can only be justified if a clinical benefit is shown. Therefore, we hypothesize that surgery of the primary tumour improves overall survival in patients with few or absent symptoms as evaluated by the treating physician and incurable stage IV CRC.

### Study design

The CAIRO4 trial is an international, multicentre, randomized, phase III study. Patients with synchronous unresectable metastatic colorectal cancer with few or absent symptoms of their primary tumour are randomized 1:1 between systemic treatment without resection of the primary tumour, and resection of the primary tumour followed by systemic treatment. Treatment according to protocol must be initiated within four weeks after randomization. The study will be conducted within the network of the Dutch Colorectal Cancer Group (DCCG) and the Danish Colorectal Cancer Group. At least 55 Dutch hospitals and 5 Danish hospitals will participate in this study, including 8 university medical centres.

### Study population

All patients who are newly diagnosed with synchronous metastatic CRC are eligible for participation in this trial when they meet the following inclusion criteria: histologically proven CRC (i.e. via colonoscopy and/or any other method) with a resectable primary tumour in situ and unresectable distant metastases without an indication for neo-adjuvant (chemo)radiation, with few or absent symptoms of the primary tumour, without prior systemic treatment for advanced disease, with age ≥ 18 years and a WHO performance status of 0, 1 or 2 [29]. Laboratory serum values obtained ≤ four weeks prior to randomization must show an adequate bone marrow function (Hb ≥ 6.0 mmol/L, absolute neutrophil count ≥ 1.5°10^9^/L, platelets ≥ 100° 10^9^/L), renal function (creatinine ≤ 1.5x upper limit of normal (ULN) and creatinine clearing ≥ 30 ml/min using the Cockcroft formula) and liver function (bilirubin ≤ 2× ULN, transaminases ≤ 3× ULN in patients without hepatic metastases or ≤ 5x ULN in patients with presence of liver metastases). Patients must have undergone a computed tomography (CT) scan in the four weeks prior to randomization. Adequate follow-up must be expected and a written informed consent must be obtained before enrolment in the study.

The following definition of few or absent symptoms is used: patients without signs or symptoms related to the primary tumour that require immediate intervention (i.e. surgery, stenting, systemic therapy or radiotherapy). The necessity of immediate intervention is left to the discretion of the treating physician. Whether or not metastases are resectable will be decided by the local multidisciplinary tumour board.

Exclusion criteria are: pregnancy, lactation, unresectable primary tumour, any condition preventing the safety or feasibility of resection of the primary tumour, second primary malignancy ≤ 5 years prior to randomization with the exception of basal cell carcinoma of the skin or adequately treated in situ carcinoma of any organ, any medical condition that prevents the safe administration of systemic treatment, previous intolerance of fluoropyrimidines, known dihydropyrimidine dehydrogenase deficiency, possibility of radical resection of all metastatic disease, uncontrolled hypertension (values ≥ 150/100 mmHg), use of > 3 antihypertensive drugs, significant cardiovascular disease < 1 year prior to randomization, chronic active infection and concurrent treatment with any other anti-cancer therapy as described per protocol. There are no exclusion criteria considering the side of metastatic disease.

Because of the higher complexity of local treatment in locally advanced rectal cancer, with patients often requiring neoadjuvant (chemo)radiation therapy and higher risk of morbidity and longer postoperative reconvalescence, these patients are excluded from the study. Patients with rectal cancer that do not require radiation therapy (i.e. rectal cancer with clinical staged T1-3 N0, extramural invasion ≤ 5 millimetres, distance to the mesorectal fascia > 1 millimetre [5]), but otherwise do meet the inclusion criteria can participate in the CAIRO4 study.

All patients who do not meet our inclusion criteria and/or refuse participation to the trial, will be asked for their consent for registration in a prospective database.

### Interventions

#### Experimental arm: surgical resection prior to systemic treatment

Patients will undergo surgical resection of the primary tumour within four weeks of randomization, followed by systemic therapy as described for the control arm. Surgical resection of the tumour should be intended as R0 resection, and may be performed by laparoscopy or open surgery, depending on the preference of the surgeon. If a complete resection of the primary tumour cannot be performed according to the operating team, a diverting stoma or entero-enterostomy is strongly advised in order to prevent complications of obstruction during follow-up. After surgical resection, patients should commence palliative systemic treatment as described for the control group when they have sufficiently recovered, but not earlier than 4 weeks after surgery.

#### Control arm: systemic treatment

Patients will receive first-line fluoropyrimidine-based chemotherapy with bevacizumab within four weeks of randomization. The exact chemotherapy schedule is to the discretion of the local investigator. Surgery of the primary tumour will only be performed when indicated by local signs or symptoms, such as obstruction or bleeding. Alternatively, if other palliative treatment options, such as endoscopic stenting or radiotherapy, are considered more suitable, either due to the nature of the symptoms or the general condition of the patient, they may be used.

#### Duration of treatment and follow-up

The systemic therapy will be continued until progression of disease or unacceptable toxicity, followed by salvage therapy at the discretion of the local investigator. In case of drug-related toxicity, this drug should be discontinued, while, if possible, the other drugs should be continued. If a treatment-free interval is considered to be in the best interest of the patients, this is allowed.

Patients will be evaluated with CT scans and a clinical encounter every 9–10 weeks for response, or in between when progression is suspected. After permanent discontinuation of therapy, patients will be followed every 3 months until progression or death.

If, at any time, the physicians involved in the treatment believe an intervention with curative intent is possible, this should be performed in any patient on the study. After radical resection systemic therapy will only be continued when advised by a multidisciplinary board of the local treatment centre. Patients will remain on-study and will be included in the analysis.

### Study objectives

The primary objective of this trial is to determine the clinical benefit in terms of overall survival of resection of the primary tumour with few or absent symptoms in patients with synchronous unresectable metastases of CRC (intention-to-treat population).

Secondary objectives include progression free survival, grade III and IV chemotherapy related toxicity, 30-day and 90-day surgery-related morbidity and mortality, quality of life, number of patients who undergo secondary surgery of initially unresectable metastases, number of patients who never receive systemic therapy in the intervention arm, interval between randomization and initiation of systemic treatment, and overall survival in patients in whom treatment according to protocol was initiated. In the control arm we will also determine the percentage of patients requiring resection of the primary tumour and the percentage of patients who require stenting or radiotherapy for symptom palliation. Furthermore a cost-benefit analysis will be performed, as well as translational research on prognostic markers.

### Endpoints

The primary study endpoint is overall survival, and the study is designed to demonstrate a difference in median overall survival of six months between both arms. Six months is believed to be the minimal difference to justify a surgical procedure in advanced patients. Although it seems a large difference between the two arms, it is in line with most published data (Table 1).

### Study assessment

All eligible randomized patients will be included in the analysis (intent-to-treat). Overall survival is estimated from the date of randomization to death from any cause. Progression free survival is defined as the time measured from the day of randomization to the date of first documented progression, or death from any cause. All adverse events, clinical and laboratory symptomatology will be graded according to NCI common toxicity criteria, version 4.0 [30].

Response will be assessed according to the RECIST criteria for evaluation of response [31]. A baseline measurement should be performed within 4 weeks prior to the start of treatment via CT scanning. A chest X-ray may be used provided in case of lung metastases if the target lesion is unidimensionally measurable and has a diameter of > 2 cm. Ultrasound and serum carcinoembryotic antigen (CEA) are not considered adequate parameters for disease evaluation.

Quality of life will be measured using the EORTC-QLQ C30 and CR38 questionnaires [32, 33], with a baseline measurement within 2 weeks prior to randomization and every six months thereafter, until the end of the study treatment.

### Statistical considerations

#### Sample size

In the control group the expected median overall survival is 13 months. In order to demonstrate a clinically relevant increase of 6 months in the experimental group, a total of 218 deaths are required (80% power, significance level 0.05). With a recruitment rate of 12 patients per month, an accrual period of 30 months and a follow-up period of 8 months, a total of 360 patients are required in order to detect a difference in median overall survival of 13 versus 19 months with a power of 80%.

#### Randomization

Patients will be randomized centrally for systemic treatment versus surgery of the primary tumour in a 1:1 allocation ratio, stratifying for number of metastatic sites (1 versus more), serum lactate dehydrogenase (LDH, normal versus abnormal), WHO performance status (0 or 1 versus 2) and institution.

#### Primary analysis

An interim analysis at a significance level of 0.001 will be performed when one third of the events have occurred. The primary analysis will be a stratified log rank test on the overall survival at a significance level of 0.049. The sample size of 360 (180 per group) is such that still 79.6% power is retained when testing a level of 0.049. Patients without recurrence and alive at the time of the analysis will be included as censored data.

Secondary parameters will be compared between the two arms using stratified log rank tests (time-to-event endpoints), chi-square tests (disease and outcome characteristics) and t-tests (e.g. quality of life). Regression analysis will be used for translational research questions.

### Ethics

The study was approved by the Central Committee of Human-related Research and by the local ethics committees of all participating centres. The CAIRO4 study is registered at clinicaltrial.gov (NCT01606098) [34]. Prior to registration written informed consent will be obtained in all patients, with a separate informed consent for the collection of samples for translational research.

An independent data monitoring committee consisting of three senior medical and surgical oncologists and a statistician, who are not involved in the study, will review the safety data on a regular basis and report their findings to the principal investigator. The principal investigator will report these findings to the ethics committee.

## Discussion

Although recent publications suggest that resection of the primary tumour in synchronous metastatic CRC patients might not be necessary, this appears to be based on feasibility and not on clinical outcome. The CAIRO4 trial is designed to analyse the role of resection of the primary tumour in unresectable metastatic CRC.

This trial has been designed to evaluate two accepted treatment strategies. Therefore, there are no specific directives regarding type of surgery and/or chemotherapy regimen. To the discretion of the local investigator, the following chemotherapy schedules are allowed: 5FU/LV, capecitabine, CAPOX, FOLFOX, FOLFIRI or CAPIRI [35–39]. In both study arms bevacizumab will be added to a fluoropyrimidine containing regimen according to the Dutch national guideline [5]. Bevacizumab is a targeted therapy, as it inhibits tumour neoangiogenesis by blocking VEGF. Tumour neoangiogenesis is a prerequisite for tumour growth. VEGF and VEGF-receptors have been implicated in this process, and have been associated with poor prognosis. Bevacizumab is worldwide accepted for use in first-line treatment of advanced CRC and its benefit has been confirmed by compelling data [40].

Considering current developments, it might be expected that there will be a decreasing percentage of patients presenting with incurable synchronous metastatic CRC. The last decades have seen major improvements in both surgical techniques as well as effectiveness of adjuvant therapy, which has led to a remarkable increase in five-year survival rates [41]. Although approximately 20-25% of patients present with synchronous distant metastatic disease, with the development of new surgical techniques, such as liver resection, pulmonary metastasectomy and hyperthermic intraperitoneal chemotherapy, an increasing number of patients are treated with curative intent [42]. In selected patients this could lead to five-year survival rates as high as 35-60% [43].

On the other hand, only a slight increase in the proportion of stage IV disease was observed in the Netherlands in the last two decades [41, 44]. This increase is probably due to an earlier and more accurate detection of distant metastases, because of more widely available and improved imaging techniques, such as magnetic resonance imaging (MRI), positron emission tomography (PET) and CT scanning. Although in many cases metastases might be better resectable when detected in an earlier stage, in others detection of unresectable metastases (i.e. in bone or distant lymph nodes) might lead to refraining from treatment with curative intent.

It can be argued that the development of nationwide screening programmes will lead to an earlier detection of CRC, and thus to a decreasing number percentage of advanced CRC [45–48]. However, randomized trials show that the specific proportion of patients with synchronous metastatic disease at diagnosis did not differ between the control group and the group that was offered screening [45–47]. Therefore, despite current developments in both detection and treatment of CRC, the issue of the best treatment strategy in patients who present with synchronous metastatic disease will still be relevant for future patients.

As of August 2012, the trial accrual for the CAIRO4 study has started in the above mentioned centres. Taking in account the time needed to implement this study in all centers, we expect that in five year time the recruitment will be completed. This trial will provide an answer to the question if resection of the primary tumour with few or absent symptoms in patients with synchronous metastatic CRC offers a clinical benefit in terms of overall survival and quality of life.

## Abbreviations

5FU/LV: Fluorouracil/leucovorin
CAPIRI: Capecitabine/irinotecan
CAPOX: Capecitabine/oxaliplatin
CEA: Carcinoembryotic antigen
CRC: Colorectal cancer
CT: Computed tomography
EORTC: European Organisation for Research and Treatment of Cancer
FOLFIRI: Leucovorin (folinic acid)/fluorouracil/irinotecan
FOLFOX: Leucovorin (folinic acid)/fluorouracil/oxaliplatin
LDH: Lactate dehydrogenase
MRI: Magnetic resonance imaging
NCI: National Cancer Institute
PET: Positron emission tomography
QLQ: Quality of life questionnaire
RECIST: Response evaluation criteria in solid tumours
ULN: Upper limit of normal
VEGF: Vascular endothelial growth factor
WHO: World Health Organisation.

## References

[1] **Dutch cancer registry** http://www.cijfersoverkanker.nl/selecties/incidentie_darmkanker/img5230620b36110

[2] van der Pool AE, Damhuis RA, Ijzermans JN, de Wilt JH, Eggermont AM, Kranse R, Verhoef C (2012). Trends in incidence, treatment and survival of patients with stage IV colorectal cancer: a population-based series. Colorectal Dis.

[3] Simmonds PC (2000). Palliative chemotherapy for advanced colorectal cancer: systematic review and meta-analysis. Colorectal Cancer Collaborative Group. BMJ.

[4] Golfinopoulos V, Salanti G, Pavlidis N, Ioannidis JP (2007). Survival and disease-progression benefits with treatment regimens for advanced colorectal cancer: a meta-analysis. Lancet Oncol.

[5] National Working Group on Gastrointestinal Cancer (2014). Colorectal Cancer – Nation-Wide Guideline, Version 3.0.

[6] Cook AD, Single R, McCahill LE (2005). Surgical resection of primary tumors in patients who present with stage IV colorectal cancer: an analysis of surveillance, epidemiology, and end results data, 1988 to 2000. Ann Surg Oncol.

[7] Eisenberger A, Whelan RL, Neugut AI (2008). Survival and symptomatic benefit from palliative primary tumor resection in patients with metastatic colorectal cancer: a review. Int J Color Dis.

[8] Kleespies A, Fuessl KE, Seeliger H, Eichhorn ME, Muller MH, Rentsch M, Thasler WE, Angele MK, Kreis ME, Jauch KW (2009). Determinants of morbidity and survival after elective non-curative resection of stage IV colon and rectal cancer. Int J Color Dis.

[9] Cirocchi R, Trastulli S, Abraha I, Vettoretto N, Boselli C, Montedori A, Parisi A, Noya G, Platell C (2012). Non-resection versus resection for an asymptomatic primary tumour in patients with unresectable stage IV colorectal cancer. Cochrane Database Syst Rev.

[10] Sorbye H, Kohne CH, Sargent DJ, Glimelius B (2007). Patient characteristics and stratification in medical treatment studies for metastatic colorectal cancer: a proposal for standardization of patient characteristic reporting and stratification. Ann Oncol.

[11] Law WL, Chan WF, Lee YM, Chu KW (2004). Non-curative surgery for colorectal cancer: critical appraisal of outcomes. Int J Color Dis.

[12] Sarela AI, Guthrie JA, Seymour MT, Ride E, Guillou PJ, O’Riordain DS (2001). Non-operative management of the primary tumour in patients with incurable stage IV colorectal cancer. Br J Surg.

[13] Scoggins CR, Meszoely IM, Blanke CD, Beauchamp RD, Leach SD (1999). Nonoperative management of primary colorectal cancer in patients with stage IV disease. Ann Surg Oncol.

[14] Peeters CF, Westphal JR, de Waal RM, Ruiter DJ, Wobbes T, Ruers TJ (2004). Vascular density in colorectal liver metastases increases after removal of the primary tumor in human cancer patients. Int J Cancer.

[15] Poultsides GA, Servais EL, Saltz LB, Patil S, Kemeny NE, Guillem JG, Weiser M, Temple LK, Wong WD, Paty PB (2009). Outcome of primary tumor in patients with synchronous stage IV colorectal cancer receiving combination chemotherapy without surgery as initial treatment. J Clin Oncol.

[16] Benoist S, Pautrat K, Mitry E, Rougier P, Penna C, Nordlinger B (2005). Treatment strategy for patients with colorectal cancer and synchronous irresectable liver metastases. Br J Surg.

[17] Damjanov N, Weiss J, Haller DG (2009). Resection of the primary colorectal cancer is not necessary in nonobstructed patients with metastatic disease. Oncologist.

[18] Karoui M, Koubaa W, Delbaldo C, Charachon A, Laurent A, Piedbois P, Cherqui D, van Tran Nhieu J (2008). Chemotherapy has also an effect on primary tumor in colon carcinoma. Ann Surg Oncol.

[19] Kuo LJ, Leu SY, Liu MC, Jian JJ, Hongiun Cheng S, Chen CM (2003). How aggressive should we be in patients with stage IV colorectal cancer?. Dis Colon Rectum.

[20] Liu SK, Church JM, Lavery IC, Fazio VW (1997). Operation in patients with incurable colon cancer–is it worthwhile?. Dis Colon Rectum.

[21] Rosen SA, Buell JF, Yoshida A, Kazsuba S, Hurst R, Michelassi F, Millis JM, Posner MC (2000). Initial presentation with stage IV colorectal cancer: how aggressive should we be?. Arch Surg.

[22] Stillwell AP, Buettner PG, Ho YH (2010). Meta-analysis of survival of patients with stage IV colorectal cancer managed with surgical resection versus chemotherapy alone. World J Surg.

[23] Venderbosch S, de Wilt JH, Teerenstra S, Loosveld OJ, van Bochove A, Sinnige HA, Creemers GJ, Tesselaar ME, Mol L, Punt CJ, Koopman M (2011). Prognostic value of resection of primary tumor in patients with stage IV colorectal cancer: retrospective analysis of two randomized studies and a review of the literature. Ann Surg Oncol.

[24] Ruo L, Gougoutas C, Paty PB, Guillem JG, Cohen AM, Wong WD (2003). Elective bowel resection for incurable stage IV colorectal cancer: prognostic variables for asymptomatic patients. J Am Coll Surg.

[25] Kaufman MS, Radhakrishnan N, Roy R, Gecelter G, Tsang J, Thomas A, Nissel-Horowitz S, Mehrotra B (2008). Influence of palliative surgical resection on overall survival in patients with advanced colorectal cancer: a retrospective single institutional study. Colorectal Dis.

[26] Tebbutt NC, Norman AR, Cunningham D, Hill ME, Tait D, Oates J, Livingston S, Andreyev J (2003). Intestinal complications after chemotherapy for patients with unresected primary colorectal cancer and synchronous metastases. Gut.

[27] Michel P, Roque I, Di Fiore F, Langlois S, Scotte M, Teniere P, Paillot B (2004). Colorectal cancer with non-resectable synchronous metastases: should the primary tumor be resected?. Gastroenterol Clin Biol.

[28] Evans MD, Escofet X, Karandikar SS, Stamatakis JD (2009). Outcomes of resection and non-resection strategies in management of patients with advanced colorectal cancer. World J Surg Oncol.

[29] Oken MM, Creech RH, Tormey DC, Horton J, Davis TE, McFadden ET, Carbone PP (1982). Toxicity and response criteria of the Eastern Cooperative Oncology Group. Am J Clin Oncol.

[30] National Cancer Institute (2009). Common Terminology Criteria for Adverse Events (CTCAE), Version 4.0.

[31] Eisenhauer EA, Therasse P, Bogaerts J, Schwartz LH, Sargent D, Ford R, Dancey J, Arbuck S, Gwyther S, Mooney M, Rubinstein L, Shankar L, Dodd L, Kaplan R, Lacombe D, Verweij J (2009). New response evaluation criteria in solid tumours: revised RECIST guideline (version 1.1). Eur J Cancer.

[32] Aaronson NK, Ahmedzai S, Bergman B, Bullinger M, Cull A, Duez NJ, Filiberti A, Flechtner H, Fleishman SB, de Haes JC, Kaasa S, Klee M, Osaba D, Razavi D, Rofe PB, Schraub S, Sneeuw K, Sullivan M, Takeda F (1993). The European Organization for Research and Treatment of Cancer QLQ-C30: a quality-of-life instrument for use in international clinical trials in oncology. J Natl Cancer Inst.

[33] Sprangers MA, te Velde A, Aaronson NK (1999). The construction and testing of the EORTC colorectal cancer-specific quality of life questionnaire module (QLQ-CR38). European Organization for Research and Treatment of Cancer Study Group on Quality of Life. Eur J Cancer.

[34] **The role of surgery of the primary tumour with few or absent symptoms in patients with synchronous unresectable metastases of colon cancer (CAIRO4)** http://clinicaltrial.gov/ct2/show/NCT01606098 10.1186/1471-2407-14-741 PMC4196118 25277170

[35] Chibaudel B, Maindrault-Goebel F, Lledo G, Mineur L, Andre T, Bennamoun M, Mabro M, Artru P, Carola E, Flesch M, Dupuis O, Colin P, Larsen AK, Afchain P, Tournigand C, Louvet C, de Gramont A (2009). Can chemotherapy be discontinued in unresectable metastatic colorectal cancer? The GERCOR OPTIMOX2 Study. J Clin Oncol.

[36] de Gramont A, Figer A, Seymour M, Homerin M, Hmissi A, Cassidy J, Boni C, Cortes-Funes H, Cervantes A, Freyer G, Papamichael D, Le Bail N, Louvet C, Hendler D, de Braud F, Wilson C, Morvan F, Bonetti A (2000). Leucovorin and fluorouracil with or without oxaliplatin as first-line treatment in advanced colorectal cancer. J Clin Oncol.

[37] Koopman M, Antonini NF, Douma J, Wals J, Honkoop AH, Erdkamp FL, de Jong RS, Rodenburg CJ, Vreugdenhil G, Loosveld OJ, van Bochove A, Sinnige HA, Creemers GJ, Tesselaar ME, Slee PH, Werter MJ, Mol L, Dalesio O, Punt CJ (2007). Sequential versus combination chemotherapy with capecitabine, irinotecan, and oxaliplatin in advanced colorectal cancer (CAIRO): a phase III randomised controlled trial. Lancet.

[38] Tournigand C, Cervantes A, Figer A, Lledo G, Flesch M, Buyse M, Mineur L, Carola E, Etienne PL, Rivera F, Chirivella I, Perez-Staub N, Louvet C, André T, Tabah-Fisch I, de Gramont A (2006). OPTIMOX1: a randomized study of FOLFOX4 or FOLFOX7 with oxaliplatin in a stop-and-Go fashion in advanced colorectal cancer–a GERCOR study. J Clin Oncol.

[39] Tournigand C, Andre T, Achille E, Lledo G, Flesh M, Mery-Mignard D, Quinaux E, Couteau C, Buyse M, Ganem G, Landi B, Colin P, Louvet C, de Gramont A (2004). FOLFIRI followed by FOLFOX6 or the reverse sequence in advanced colorectal cancer: a randomized GERCOR study. J Clin Oncol.

[40] Petrelli F, Borgonovo K, Cabiddu M, Ghilardi M, Lonati V, Maspero F, Sauta MG, Beretta GD, Barni S (2013). FOLFIRI-bevacizumab as first-line chemotherapy in 3500 patients with advanced colorectal cancer: a pooled analysis of 29 published trials. Clin Colorectal Cancer.

[41] van Steenbergen LN, Elferink MA, Krijnen P, Lemmens VE, Siesling S, Rutten HJ, Richel DJ, Karim-Kos HE, Coebergh JW, Working Group Output of The Netherlands Cancer R (2010). Improved survival of colon cancer due to improved treatment and detection: a nationwide population-based study in The Netherlands 1989–2006. Ann Oncol.

[42] Lochan R, White SA, Manas DM (2007). Liver resection for colorectal liver metastasis. Surg Oncol.

[43] Verhoef C, de Wilt JH, Burger JW, Verheul HM, Koopman M (2011). Surgery of the primary in stage IV colorectal cancer with unresectable metastases. Eur J Cancer.

[44] Lemmens V, van Steenbergen L, Janssen-Heijnen M, Martijn H, Rutten H, Coebergh JW (2010). Trends in colorectal cancer in the south of the Netherlands 1975–2007: rectal cancer survival levels with colon cancer survival. Acta Oncol.

[45] Hardcastle JD, Chamberlain JO, Robinson MH, Moss SM, Amar SS, Balfour TW, James PD, Mangham CM (1996). Randomised controlled trial of faecal-occult-blood screening for colorectal cancer. Lancet.

[46] Kronborg O, Fenger C, Olsen J, Jorgensen OD, Sondergaard O (1996). Randomised study of screening for colorectal cancer with faecal-occult-blood test. Lancet.

[47] Lindholm E, Brevinge H, Haglind E (2008). Survival benefit in a randomized clinical trial of faecal occult blood screening for colorectal cancer. Br J Surg.

[48] Mandel JS, Bond JH, Church TR, Snover DC, Bradley GM, Schuman LM, Ederer F (1993). Reducing mortality from colorectal cancer by screening for fecal occult blood. Minnesota Colon Cancer Control Study. N Engl J Med.

[CR49] The pre-publication history for this paper can be accessed here:http://www.biomedcentral.com/1471-2407/14/741/prepub

